# Augmenting Around-Device Interaction by Geomagnetic Field Built-in Sensor Utilization

**DOI:** 10.3390/s21093087

**Published:** 2021-04-28

**Authors:** Sandi Ljubic, Franko Hržić, Alen Salkanovic, Ivan Štajduhar

**Affiliations:** 1 Faculty of Engineering, University of Rijeka, Vukovarska 58, HR-51000 Rijeka, Croatia; franko.hrzic@riteh.hr (F.H.); alen.salkanovic@riteh.hr (A.S.); istajduh@riteh.hr (I.Š.); 2 Center for Artificial Intelligence and Cybersecurity, University of Rijeka, R. Matejcic 2, HR-51000 Rijeka, Croatia

**Keywords:** Around-Device Interaction (ADI), geomagnetic field sensor, touchless interaction, magnetic field fingerprinting, pointing, neural networks

## Abstract

In this paper, we investigate the possibilities for augmenting interaction around the mobile device, with the aim of enabling input techniques that do not rely on typical touch-based gestures. The presented research focuses on utilizing a built-in magnetic field sensor, whose readouts are intentionally affected by moving a strong permanent magnet around a smartphone device. Different approaches for supporting magnet-based Around-Device Interaction are applied, including magnetic field fingerprinting, curve-fitting modeling, and machine learning. We implemented the corresponding proof-of-concept applications that incorporate magnet-based interaction. Namely, text entry is achieved by discrete positioning of the magnet within a keyboard mockup, and free-move pointing is enabled by monitoring the magnet’s continuous movement in real-time. The related solutions successfully expand both the interaction language and the interaction space in front of the device without altering its hardware or involving sophisticated peripherals. A controlled experiment was conducted to evaluate the provided text entry method initially. The obtained results were promising (text entry speed of nine words per minute) and served as a motivation for implementing new interaction modalities. The use of neural networks has shown to be a better approach than curve fitting to support free-move pointing. We demonstrate how neural networks with a very small number of input parameters can be used to provide highly usable pointing with an acceptable level of error (mean absolute error of 3 mm for pointer position on the smartphone display).

## 1. Introduction

Direct touch (tapping) and touch gestures (dragging and swiping) still represent predominant interaction modalities for contemporary mobile devices. In addition to the nowadays less utilized stylus/pen, the human finger serves as the primary input device for smartphone and tablet gadgets. Touch-based interaction, on the other hand, comes with specific drawbacks due to the limited form factor of touchscreen devices and the effects of the well-known fat finger syndrome. For example, when using a smartphone with one hand, the touch gestures become more difficult and less comfortable to perform. Popular wearable devices, like smartwatches, have even more limited input capabilities; therefore, the suitability of touch-based interaction can be questioned in such a context as well.

To overcome the shortcomings of touch-based interaction, alternative modalities that rely on sensory feedback have been implemented. Many solutions aim to enhance the interactivity with a mobile device by making use of built-in sensors. For example, an accelerometer can be used to detect smartphone tilting, and the corresponding movements (pitch, roll, and yaw) can then be interpreted as input commands. This means that specific tasks, such as adjusting the screen orientation, navigating the user interface, or controlling a mobile game, can actually be done without touching the device display.

Many research efforts are centered on augmenting the input space around a device. The corresponding concept, called Around-Device Interaction (ADI), assumes solutions that are based on detecting movement gestures or environmental changes in the vicinity of a mobile device. According to Jones et al. [1], ADI can achieve performances comparable to touch interaction while providing a larger operating area. One of the earliest manifestations of this idea refers to SideSight [2]. A prototype fitted with infrared proximity sensors allows the device’s surrounding surface to mimic the multi-touch interaction space. Furthermore, authors of AirPanes [3] and AD-Binning [4] utilized infrared markers in conjunction with motion capture cameras to provide in-air gestures around the device. Additionally, as shown by Schmieder et al. [5], a front-facing camera can be successfully used to capture finger movements performed above the smartphone. The built-in microphone can also be particularly useful, as it can support typical voice-enabled interaction, as well as some less conventional smartphone applications. Namely, Wang et al. [6] proved that finger-tapping on a wooden table creates an authentic audio signal that can be localized and subsequently interpreted as both meaningful and intentional text input.

Google’s project Soli [7,8] demonstrates the rising popularity of ADI and the importance of sensory-based input in ubiquitous computing. It presents a motion-tracking sensing technology based on a miniature radar. Both the thumb and index finger can make fine gestures that can be detected and used within a 3D gesture interaction language. Soli supports units of interaction that can build “complex interactive experiences that go beyond touch and voice”. Google’s Pixel 4 was the first smartphone model with integrated Soli microchip, allowing users to skip songs, snooze alarms, and silence phone calls, without actually touching the device.

When it comes to the general acceptance of ADI, Ahlström et al. [9] revealed that, in such a context, gestures closer to the device are more acceptable. According to the related research, most end-users would gladly use ADI if the corresponding gestures were done within a critical point distance of 30 cm from the device.

The main motivation of our work was to explore the possibilities for ADI enhancement by using the smartphone’s geomagnetic field built-in sensor exclusively. Instead of relying on multiple sensor fusion, we demonstrate how a typical magnetometer can be utilized for touchless interaction with the mobile device. The key contributions of this paper are gathered around the following activities:

- augmenting ADI without the need for a specific hardware accessory (only a simple permanent magnet is used),
- demonstrating different approaches for magnetometer readout utilization, ranging from magnetic field fingerprinting to the solution based on neural networks (NNs),
- providing proof-of-concept applications that rely on magnet-based interaction, involving text entry (based on discrete magnet positioning), and free-move pointing (based on continuous magnet movement),
- avoiding implementation complexity without affecting the usability, by making use of a simple fingerprinting method and neural network (NN) design with a limited number of parameters,
- providing initial evaluations of the proof-of-concept applications.

### Related Work

Numerous research efforts have been focused on utilizing the geomagnetic field sensor to facilitate ADI for handheld devices. Magnets have many advantages over other interaction accessories, including low cost, durability, a broad variety of shapes, and are completely passive—they require no power. These advantages were recognized by many researchers who utilized magnets in various applications that required enhanced user interaction. Magnets may be used to improve usability and reduce screen occlusion by expanding interactivity beyond the device screen. For instance, Bianchi et al. [10] developed magnetic appcessories enabling smartphone interaction without touching the screen, thereby mitigating the occlusion issue. Furthermore, MagGetz [11] solution enables tangible interaction on and around handheld devices by making use of adaptable passive control magnetic widgets. The corresponding toolkit provides various controllers, such as push-buttons, sliders, toggle switches, rotational wheels, etc.

One of the pioneer applications that utilized magnets for improving interaction possibilities is Abracadabra [12]. The authors of Abracadabra employed an external magnetic field provided by a neodymium magnet attached to the user’s index finger as an input technique to control the small wristwatch-like devices. Ketabdar et al., in their work, created various applications based on the gestures formed by moving the magnet (in the shape of rod or ring) in a space around the device [13]. The formed gestures invoke different actions concerning the application they are paired with. The solutions based on this approach are: MagiTact [13] (3D gestures used for interacting with the music player, maps, and photo gallery application), MagiWrite [14] and MagiThings [15] (3D gestures used to insert digits), MagController [16] (magnet-based actions to manipulate objects in 3D space), MagiSign [17] (authentication strategy based on 3D magnetic signatures), and MagiGuitar [18] (allows in-air gestures to mimic playing the guitar).

Gesture detection based on the magnetometer readouts has also been shown helpful in cases where a mobile device can not be easily accessed. Smus and Riederer [19] dealt with the Google Cardboard magnet input to interact with mobile virtual reality (VR) application. They opted for such a solution because the smartphone’s touchscreen was concealed within the VR enclosure.

Another type of application leverages the embedded magnetometer for providing textual input to smartphone devices. Solutions such as MagBoard [20] and MagStroke [21] utilize custom-printed keyboard to detect keystrokes made while wearing specially designed ring magnets. The detected keystrokes are transferred as textual inputs to the underlying application on the mobile device.

Several authors introduced spatial mobile device interaction using a specially developed stylus. Abe et al. [22] proposed two different input techniques: Non-electrical (where a permanent magnet is attached at the tip of the stylus) and electrical (where the electromagnet is used instead). MagPen [23] is another solution introducing a magnetic-driven pen controller that operates both on and around handheld devices. In contrast to MagPen, TMotion [24] empowers the magnet usage accompanied with the inertial measurement unit (IMU), providing the embedded magnet’s current orientation. This way, tracking the 3D position of the permanent magnet can be performed without the assistance of machine learning algorithms.

In most of the mentioned research papers [13,17,21,23,24], permanent magnets with specific forms (e.g., ring) or within the specific accessory (e.g., pen) were used, and their movement was detected by utilizing artificial NNs. Furthermore, in many other ADI-based research efforts, the various forms of NNs are utilized to either detect interaction patterns or classify the gestures. For instance, Sun et al. [25] utilized a convolutional neural network (CNN) to extract features and predict the lip-sync commands. Mayer et al. [26] have also used CNN, however to detect finger orientation based on the off-the-shelf capacitive touchscreen features. The big breakthrough was made by the introduction of mobilenets [27], which became the backbone of many vision-based applications in the mobile domain.

In our study, from the NN perspective, the task of predicting the magnet position is not computationally demanding and is characterized by a limited set of input features. Hence, we had followed the intuition of related work (mentioned before) and utilized the standard feed-forward NN. When choosing the NN topology, we considered the trade-off between NN complexity and its performance. More advanced NNs [28,29] usually have considerably more weights and are more computationally demanding. Therefore, we planned to start with the simpler forms of NNs and, only if necessary, incrementally build the more complex models utilizing convolutional or recurrent blocks. In the end, the feed-forward NN showed to be a suitable solution for the target use case.

## 2. Materials and Methods

**Figure 1:**
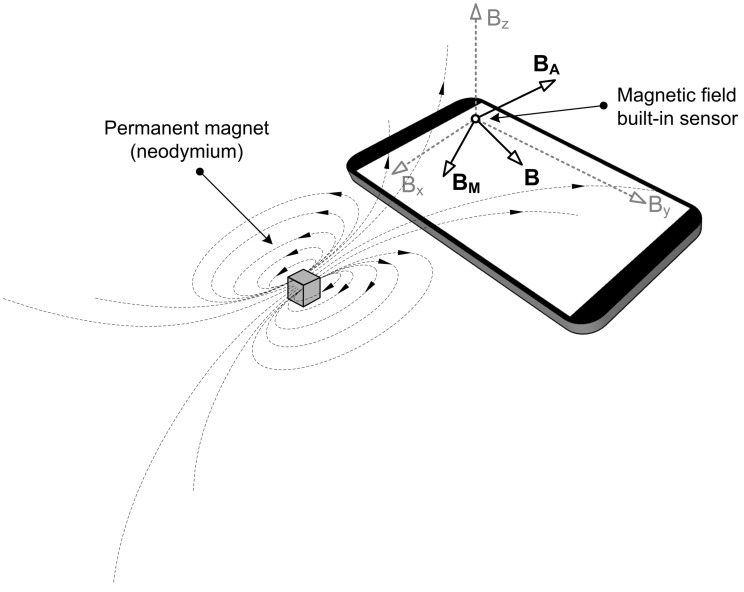
The impact of a permanent magnet put in the vicinity of a mobile device.

In the context of augmenting ADI, the key concept behind magnetic field manipulation is that any external magnetic source affects the readouts given by a nearby geomagnetic field sensor. Figure 1 depicts this fundamental principle.

The embedded geomagnetic field sensor (also known as a compass or magnetometer) determines the magnetic field vector *B* by measuring magnetic flux density along three axes. In the majority of cases, this vector reflects the Earth’s magnetic field in the given position. However, as the authors of MagiWrite [14] show, the sensor can be influenced by specific sources of external magnetic noise. Hence, placing a strong permanent magnet (such as neodymium) near a mobile device will significantly alter the surrounding magnetic field. Figure 1 provides an example of such a setup, in which the resultant vector *B* can be represented by two components. Namely, while the first component ($B_{A}$) corresponds to the ambient magnetic field with inherent environmental noise, the second one ($B_{M}$) represents the effect induced explicitly by the permanent magnet placed in front of the mobile device.

In view of the above, we utilized the following three-step procedure to calculate the effect exclusively induced by the permanent magnet:

1. measure the ambient magnetic field in a given setup;
2. repeat the measurement with a permanent magnet placed near the mobile device;
3. subtract the ambient magnetic field from the values obtained in the second measuring.

In all parts of our research, we used an N45-grade nickel-plated neodymium magnet cube with dimensions 12×12×12 mm and magnetic flux density of 0.53 T on the surface of the cube. Regarding the mobile device, Samsung Galaxy S9+ (SM-G965F) smartphone (Samsung Electronics Co., Ltd., Suwon, Korea), running the Android 9 (Pie) operating system, was used in all experimental settings. According to iFixit [30], this device model incorporates the AKM AK09916 magnetometer microchip (Asahi Kasei Microdevices Corporation, Tokyo, Japan). Based on the corresponding datasheet [31], the microchip is composed of three magnetic sensors for detecting terrestrial magnetism in the X-axis, Y-axis, and Z-axis. It additionally integrates a sensor driving circuit, signal amplifier chain, and an arithmetic circuit for processing the signal from each sensor. This magnetometer has a measurement resolution of 0.15 μT and a limitation for the measurement range that the sum of absolute values of each axis should be smaller than 4912 μT. When the magnetic field exceeds this limitation, measurement data are considered erroneous. In order to avoid this effect, called Magnetic Sensor Overflow (MSO), the external magnet should not be placed too close to the magnetometer.

### 2.1. Invoking Discrete Commands: Magnetic Field Fingerprinting

The properties, position, and orientation of the permanent magnet will have a synergistic effect on the sensor measurements. Assuming that the magnetic properties (magnetic flux density) and orientation remain constant, magnetometer readings can be used to localize the permanent magnet in front of the smartphone. The corresponding concept is called magnetic field fingerprinting.

**Figure 2:**
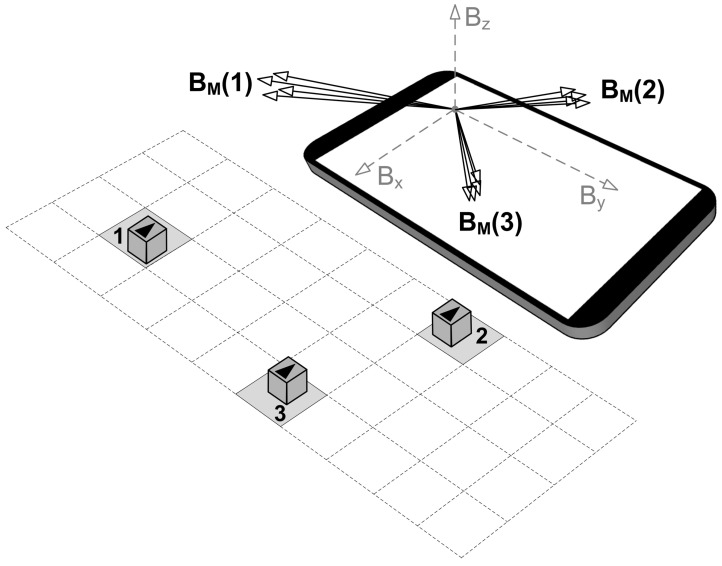
Magnetic field fingerprinting—mapping locations of the permanent magnet with the corresponding sensor readouts.

Figure 2 illustrates a hypothetical case of magnetic field fingerprinting, wherein three square-shaped zones in front of the mobile device are chosen to represent particular input commands. The permanent magnet should be moved within the limits of the appropriate zone during the calibration phase of the magnetic field fingerprinting. Such a movement will trigger different sensor outputs, and the corresponding vector range can be registered as a location-specific feature. For example, in Figure 2, vector ranges $B_{M}$(1), $B_{M}$(2), and $B_{M}$(3) correspond to the selected locations 1, 2, and 3, respectively. The magnetic field fingerprints collected in this way can be permanently saved and later used for magnet localization. As for the localization phase, the readings of the sensor are constantly monitored in real-time and compared with the previously registered magnetic field fingerprints. In case a match is detected, the location of the magnet is determined, and the appropriate application command can be invoked.

#### 2.1.1. Proof-of-Concept Application: Text Entry on a Paper Mock-Up Keyboard

Interaction zones in front of the device should be selected in such a way as to avoid overlapping magnetic fingerprints. However, this does not rule out the possibility for the selected locations to be close to each other. To support such an argument, we decided to design an interface with densely spaced elements. Hence, for our proof-of-concept application, we opted for contact-less text entry by using a permanent external magnet and a QWERTY-based paper mock-up keyboard. QWERTY is an adjective used to describe the design of the standard Western (or Latin-based) keyboard layout. The name corresponds to the order of the first six letters on the top left row of the keyboard.

**Figure 3:**
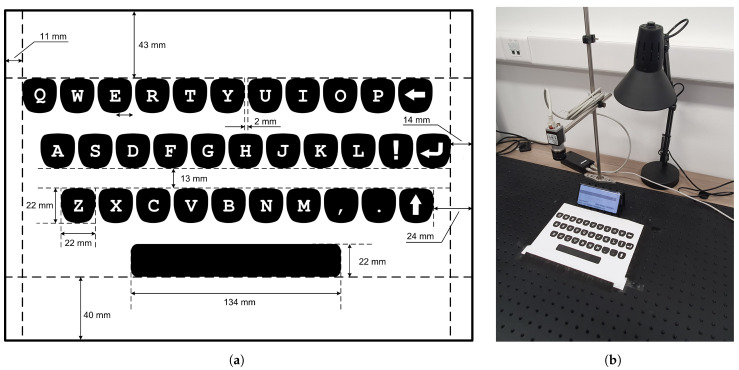
(**a**) Virtual keyboard layout used for text entry method enabled by magnet-based ADI. (**b**) Experiment setup for virtual keyboard magnet fingerprinting.

We printed a virtual keyboard, encompassing a standard QWERTY character layout, on a regular A4 paper. The geometry of the designed mock-up keyboard is shown in Figure 3a.

The paper/keyboard was placed on a flat surface in front of the smartphone. The smartphone itself was set in landscape orientation and leaned on an improvised structure made of plastic material. We opted for such a setup (shown in Figure 3b) in order to easily stabilize the mobile device in the same position, as well as to provide better visibility of the screen within the user interaction context. In the given setting, the top row of the virtual keyboard was approximately 5 cm away from the bottom part of the mobile device. This proved to be far enough away from the zone of the MSO effect. The camera located above the virtual keyboard was not used in this part of the research.

We developed an Android application that enables both magnetic field fingerprinting and related magnet localization. At the beginning of the calibration procedure, an ambient magnetic field has to be recorded to cancel its effect. Then, for each character zone, a permanent magnet has to be moved within the given boundaries for a couple of seconds. Following this, all characteristic values of $B_{x}$, $B_{y}$, and $B_{z}$ (having the ambient magnetic field subtracted) can be registered. Magnetic field fingerprints thus obtained, along with the corresponding characters, are stored in the application’s dedicated memory (Android Shared Preferences). It took no more than 10 min to calibrate the character layout from Figure 3a.

Once the fingerprinting procedure has been completed, the same mobile application can be immediately used for text entry demonstration. For any given setup in which the text entry is intended to be performed, whether experimental or informal, only the following preparatory actions need to be done beforehand:

- the mobile device and the virtual keyboard must be set up in the same way as in the fingerprinting-based calibration;
- the magnetic field without the presence of a permanent magnet must be measured and registered (in order to filter out the ambient effect).

The fingerprint matching is performed at the highest possible sampling rate of the used sensor along with the ongoing text entry task. This is achieved via SENSOR_DELAY_FASTEST setting in the mobile application. This setting applies to Android sensor programming, in which developers can adjust the readout frequency of a particular sensor by choosing from several predefined configurations. Namely, if we want the sensor to read the geomagnetic field values with the highest possible frequency, then SENSOR_DELAY_FASTEST must be selected and appropriately used in the related programming code. For the device used in our study, the frequency obtained in this way was ≈100 Hz. Within the provided magnet-based text entry method, we additionally incorporated two time-based features:

- Dwell time is a parameter used to avoid unnecessary character entries. It represents the minimum time during which it is necessary to keep the magnet within the particular zone in order to enter a matching character. The default value is set to 150 ms.
- Delay time is used for enabling consecutive input of the same character. Hence, this is the time we are required to retain the permanent magnet within a character zone in order to reactivate the same fingerprint. The default value is set to 700 ms.

We provide a Supplementary Video File (Video S1), which demonstrates the text entry task being executed via magnet-based ADI.

#### 2.1.2. Empirical Evaluation of Magnet-Based Text Entry

Implementation of the described magnet-based text entry method was analyzed by conducting a controlled experiment involving 28 participants. All experiment activities were carried out while respecting epidemiological measures due to the COVID-19 pandemic.

**Figure 4:**
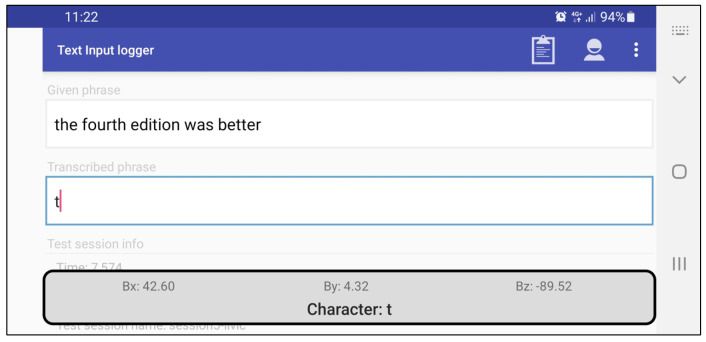
Text Input Logger—Android application used for text entry empirical research.

For testing purposes, the proposed magnet-based text entry method was implemented as an Android Input Method Editor (IME) service. This service was subsequently installed and registered as a default input method on the target device, allowing for its usage within any mobile application that requires text entry. We integrated the previously described fingerprinting calibration procedure within the IME preference activity. This, in turn, enabled us to easily set up the magnet-based text entry in any given location. Furthermore, in empirical testing, we used our own Android application (called Text Input Logger) for gathering standard text entry metrics [32], of which words per minute (WPM) and total error rate (TER) were considered as the most significant ones. The logging application implements transcription-based text entry tasks, meaning that each trial requires rewriting a displayed text phrase randomly selected from a 500 instances set developed by MacKenzie and Soukoreff [33]. Figure 4 shows the snapshot of the Text Input Logger when magnet-based IME service is active. Instead of providing IME service without any visible layout, we opted to present some sort of meaningful feedback to the end-user. Therefore, while the permanent magnet is moved in front of the mobile device, current magnetometer readout values are displayed on the smartphone screen, along with the detected character.

Twenty-eight subjects have been engaged in the empirical research. The participants’ age varied from 21 to 38 years, with an average of 25.8 years (σ=5.7). To get acquainted with the proposed magnet-based text entry method, as well as with timing constraints (i.e., dwell time and delay time), the participants were engaged in a short practice session before the actual experiment. In the actual experiment, the users were instructed to enter ten different text phrases “as quickly as possible, as accurately as possible”, using only a permanent magnet in front of the landscape-oriented smartphone device. All network-based services on the smartphone were turned off during the experiment.

Along with the initial quantitative evaluation, we wanted to get early subjective impressions of the proposed solution. Therefore, after completing all text entry tasks, the participants were asked to complete a post-study questionnaire based on the rating part of the NASA-TLX [34]. In practice, many researchers skip the weighting step of the NASA-TLX standard, which reduces the amount of time needed to administer the test, and analyze the raw TLX responses only (Raw-TLX format) [35]. Since we did not conduct longitudinal training, nor in-situ comparison with similar solutions, we opted for such a “quick-and-light” approach embodied by Raw-TLX. Moreover, we further deviated from the TLX standard by intentionally omitting one TLX factor (temporal demand) because it did not seem too important in our context. Hence, individual opinions about perceived workload had to be estimated using standard 21-point rating scales for five TLX factors: Mental demand, physical demand, frustration, performance, and overall effort.

### 2.2. Free-Move Pointing

Following the users’ positive feedback and promising results from the first part of our research (Section 3.1), we wanted to further enhance magnet-based ADI by enabling free-move pointing operation.

**Figure 5:**
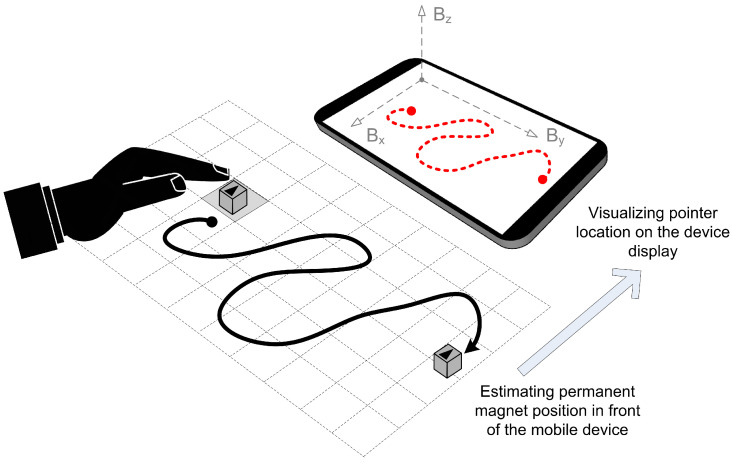
The concept of free-move pointing operation enabled by magnet-based interaction.

In the application of free-move pointing, the goal is to operate a pointer on the smartphone display by moving the permanent magnet, which continuously affects the smartphone’s geomagnetic field sensor. In other words, the main objective is to find a continuous function-relation between the permanent magnet position and the related point location on the smartphone screen. This goal is illustrated in Figure 5.

To create a practical application of free-move pointing, we have set several constraints that would reduce the moving space of the permanent magnet and make the pointer control more intuitive. The constraints are as follows:

- the magnet must be moved inside the grid, i.e., inside the virtual pane visualized on a piece of paper,
- smartphone must be set at the fixed location, relative to the depicted grid,
- the magnet orientation should be kept the same at all times; otherwise, the effect of the imposed magnetic field diverges, and the calculated relation between magnet position and pointer location can become invalid.

By applying the constraints mentioned above, the permanent magnet can be used as a smartphone’s accompanying pointing device (e.g., “computer mouse”) on a virtual pane that can be treated as a “mouse pad”. However, the function that translates the magnet position (being derived from geomagnetic field sensor readings $B_{x}$, $B_{y}$ and $B_{z}$) to the pointer location on a smartphone display ($P_{x}$, $P_{y}$) must be found. For achieving this, we utilized two different approaches:

- Curve fitting approach has emerged as an idea based on the results from the first part of the research. Namely, this approach considers the magnetic field values of the zones that reside on a straight trajectory.
- NNs approach relies on the model’s ability to learn complex functions based on the input and output data.

In the following two subsections, both approaches will be discussed in detail.

#### 2.2.1. Curve Fitting Approach

The curve fitting localization procedure is based on the premise that the position of the moving magnet can be determined with respect to the magnetic fingerprints that reside on the respective movement trajectory. In other words, if there are known magnetic field values for several points on the straight path of the magnet, we want to find a relation by which we can predict the magnetic field values for all points on that path. From a mathematical perspective, we aim to construct a curve or mathematical function that best fits a series of data points.

**Figure 6:**
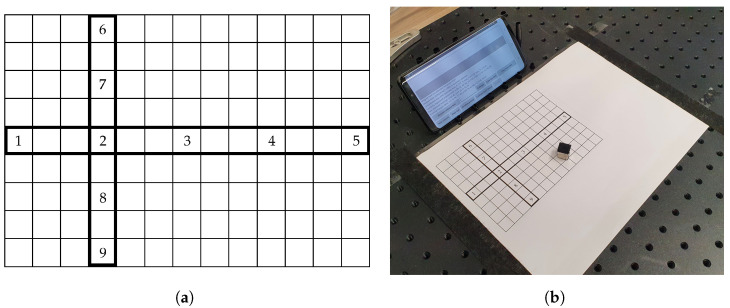
(**a**) Canvas layout used for testing the curve fitting approach. (**b**) Experiment setup for testing the curve fitting approach.

For testing the mentioned premise, we designed a simple canvas as a dedicated space for moving the magnet in front of the mobile device. The canvas is visualized as a 13×9 grid, having dimensions 156×108 mm. Therefore, each grid cell occupies exactly 12×12 mm, which corresponds to the dimensions of the side of the used cube-shaped permanent magnet. We selected and specifically highlighted two straight lines (i.e., magnet movement paths) within the designed canvas: One horizontal and one vertical. We selected and marked five distinct zones on each path, in which we planned to perform magnetic field measurements. The canvas and the corresponding experiment setup are presented in Figure 6a,b, respectively.

In order to simplify the experiment in this initial phase, we deliberately opted for proof-of-concept, which involves only two straight trajectories. As a result, data acquisition was a rather straightforward process. Each zone was fingerprinted by the mean values of magnetic field readings. More specifically, we registered mean values of $B_{x}$, $B_{y}$, and $B_{z}$, provided by the magnetometer, for nine selected positions within the canvas.

For instance, if we concentrate on the horizontal trajectory (from left to right), we can see that magnetic field values are now known for five points (1–2–3–4–5). These points reside on specific positions relative to the trajectory length (0–0.25–0.5–0.75–1). We utilized obtained magnetic field values in these 5 points for curve fitting. An analogous procedure was used for the vertical trajectory, for the point set (6–7–2–8–9), looking from top to bottom. Third-degree polynomials showed to be sufficient for achieving very high goodness of fit. All obtained curve fitting models (for $B_{x}$, $B_{y}$ and $B_{z}$—both for horizontal and vertical trajectory) are presented in Section 3.2.

**Figure 7:**
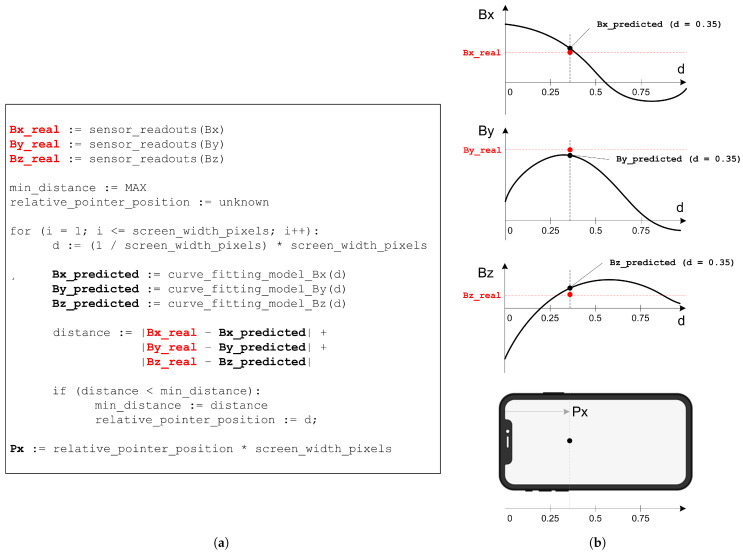
(**a**) Algorithm (pseudo-code) for determining the pointer x-axis location on the smartphone screen, by making use of the curve fitting approach. (**b**) Visualization of the implemented localization concept.

When it comes to the localization of a moving magnet, the obtained models are used to find the best candidate position for which the predicted magnetic field values are closest to the real ones. The corresponding algorithm embedded in the Android mobile application, along with the visualization of the implemented localization concept, is presented in Figure 7.

The feasibility of the curve fitting approach (for providing free-move pointing) depends on the discretization density of the given canvas. A better pointer control can be expected if more horizontal and vertical trajectories are modeled via curve fitting. However, a large number of curve fitting models increases the complexity of the localization algorithm. In such a case, the best candidate position has to be found in real-time within a larger search space. For demonstration purposes, we subsequently obtained ten curve fitting models targeting five horizontal and five vertical trajectories.

#### 2.2.2. NN Approach

As mentioned before, we are trying to solve the same problem using the NN approach. This involves finding a function that translates geomagnetic field readings from a smartphone sensor ($B_{x}$, $B_{y}$, and $B_{z}$) to the pointer position ($P_{x}$, $P_{y}$) on the smartphone display. In order to train a NN, we had to create a representative dataset first.

To generate a dataset and be precise in deducing the correct magnet position, we have developed a specially adapted object detection application. We set the camera to capture the bird’s eye-view perspective of the 15.5×10 cm canvas grid in which the magnet will be moved. First, the canvas position needs to be detected on the camera image because the magnet position will be determined in relation to its dimensions. Once the magnet is detected as a square within the camera frame, we obtain the corresponding center point $\left(M_{x},M_{y}\right)$. The magnet position $\left(M_{x},M_{y}\right)$ is then translated to the grid coordinate system, assuming that the grid’s origin $(G_{x0},G_{y0}$) represents the top-left corner of the canvas. Coordinates of its bottom-right corner $\left(G_{xn},G_{yn}\right)$ have to be determined as well. This way, we have defined the magnet position inside the grid with known dimensions. From this point on, we can obtain the scale-invariant magnet position ($P_{x}^{\prime }$, $P_{y}^{\prime }$), by involving the grid’s dimensions:

(1) $$\begin{array}{c} P_{x}^{\prime }=\frac{M_{x}-G_{x0}}{G_{width}}=\frac{M_{x}-G_{x0}}{G_{xn}-G_{x0}},\\ P_{y}^{\prime }=\frac{M_{x}-G_{x0}}{G_{height}}=\frac{M_{y}-G_{y0}}{G_{yn}-G_{y0}}. \end{array}$$

After the magnet position is obtained in the desired format, the pointer’s position ($P_{x}$, $P_{y}$) on the smartphone display can be easily determined. Namely, it is calculated by multiplying $P_{x}^{\prime }$ and $P_{y}^{\prime }$ with the device display width and height, respectively.

The position of the grid is obtained by analyzing the image taken by the camera. In particular, the largest rectangle from the corresponding A4 paper sheet must be found within the image. To find the largest rectangle, artifacts are removed from the gray-scale image by binarizing the image using the threshold value 100. This threshold value was determined empirically in a search space of values ranging from 10 to 250 with an increment of 10. Finally, the rectangles are extracted by utilizing the algorithm proposed by Suzuki et al. [36]. The same principle was used to extract the magnet position from the image captured by the camera. The binarization threshold value for magnet detection was empirically determined and set to 40. Once the magnet contours were found, they were supplemented to form the minimum bounding rectangle. The minimum bounding rectangle approximation was made by utilizing rotating calipers algorithm [37]. To check if the magnet’s orientation during the data acquisition is correct, we have implemented the magnet angle-check algorithm. If the detected bounding box of the magnet is tilted by more than $5^{\circ }$, the rectangle will become colored in red, thus signalizing its invalid orientation. Otherwise, the color of the magnet would be green.

**Figure 8:**
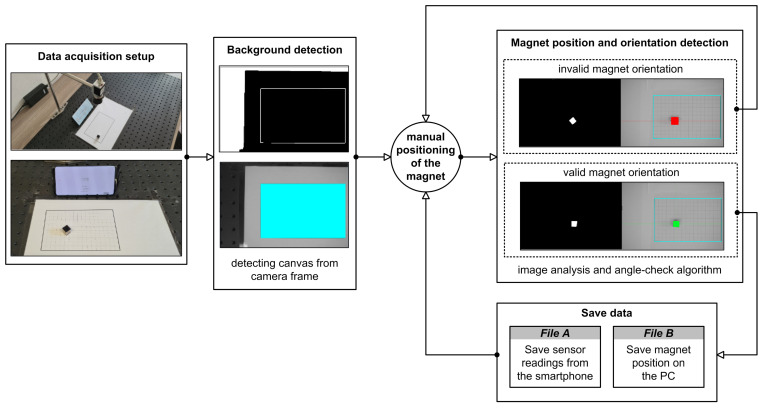
The process of dataset acquisition for the NN approach.

When the magnet position was successfully detected, we would save it to a file. At the same time, we monitored the geomagnetic field sensor readings for 3 s for each valid magnet position. This way, we obtained two paired data files with corresponding measurements: The first file containing sensors readings from the smartphone and the second file containing magnet position detected via a camera connected to the PC. Hence, while the magnet’s ground-truth location is obtained by making use of image analysis algorithms, the corresponding magnetic field values are registered via smartphone in a fixed position. Each measure required manual placement of the magnet within the canvas grid. We have decided to cover the whole grid by uniformly moving the magnet along the x-axis and y-axis of the grid. The data acquisition process resulted in 350 measurements in total. The described process is illustrated in Figure 8.

Because the described data acquisition process can be time-consuming, we opted to generate a relatively small number of data instances. To evaluate the NN model performance, we used seven-fold cross validation [38]. This resulted in a fold division where the fold training set consisted of 300 instances, while the fold test set consisted of 50 instances. The average mean squared error (MSE) and mean absolute error (MAE) of the seven folds are taken as the evaluation metrics of the tested NN topologies. MSE and MAE are calculated using the following expressions:

(2) $$\begin{array}{c} MSE=\frac{1}{n}\sum_{i=1}^{n}{\left(Y_{i}-{\hat{Y}}_{i}\right)}^{2},\\ MAE=\frac{1}{n}\sum_{i=1}^{n}\left|Y_{i}-{\hat{Y}}_{i}\right|, \end{array}$$

where *n* represents the number of data instances, *Y* is the true value, and $\hat{Y}$ is the predicted value.

We planned to apply NNs on a smartphone having limited hardware resources. In such a context, the NN must not consume too much random access memory, and needs real-time inference, consequently limiting the possible number of model parameters. Namely, too many parameters will result in slow performance on the smartphone.

As a starting point in the NN design, we have used the work of Ketabdar et al., who utilized a permanent magnet for enabling in-air signatures [17]. They have used 22 element feature vectors calculated from the geomagnetic field values as the input of the multi-layer NN. In our case, we have only three input parameters ($B_{x}$, $B_{y}$, $B_{z}$), along with two output parameters ($P_{x}^{\prime }$, $P_{y}^{\prime }$). First, we have experimented with one NN that estimates both scale-invariant values, $P_{x}^{\prime }$ and $P_{y}^{\prime }$, at the same time. However, we obtained much better results when training one NN for each parameter separately, using the same model topology. We believe that the joined cost function for the NN that estimates both parameters simultaneously creates a trade-off, resulting in estimating one parameter much better than another.

When choosing the NN topology, we performed a grid search. First, we would choose a number of dense hidden layers between 4, 5, and 6. Second, we would determine the number of neurons in each hidden dense layer between 512, 256, 128, and 64. The output layer was always only one neuron. The activation function in all layers was ReLU, except the last one where we utilized the sigmoid function [39,40]. Furthermore, as a regularization method, we have used dropout—each dense layer had a drop probability of 0.5 [41]. The initializer of dense layer weights was normal distribution having 0 mean and standard deviation 0.02. As for the optimizer, we have chosen Adam with a learning rate α=0.001 for all tested models. Namely, with a given learning rate, all tested models converged relatively fast, in under 10 min. The loss function used in training was MSE, while evaluation was done using MSE and MAE metrics.

## 3. Results

In this section, we present the results of all parts of our empirical research. We want to draw the reader’s attention to the fact that there are three Supplementary Video Files Available (Videos S1–S3) that demonstrate our proof-of-concept applications. Each video file corresponds to a specific part of the research: Magnet-based text entry, free-move pointing along the straight line (curve fitting approach), and free-move pointing based on the NN approach.

### 3.1. Magnet-Based Fingerprinting and Text Entry

**Figure 9:**
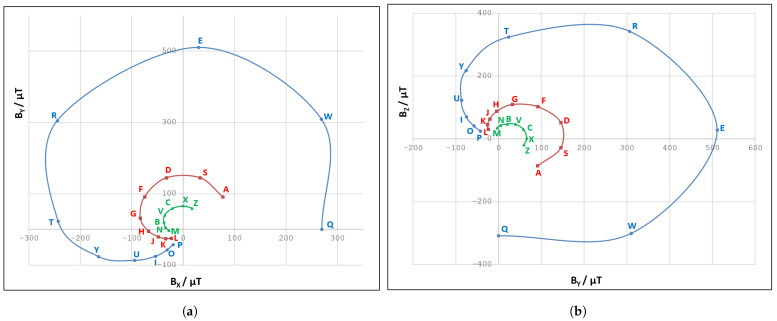
(**a**) The results of magnetic field fingerprinting on a virtual keyboard mockup (in *B_x_*/*B_y_* plane). (**b**) The same results plotted in *B_y_*/*B_z_* plane.

We find the obtained magnetic fingerprints of the letter zones within the paper mock-up keyboard rather interesting. The corresponding magnetic field values are visualized in Figure 9a,b, via $B_{x}/B_{y}$ and $B_{y}/B_{z}$ plots, respectively. For the sake of simplicity, only letter fingerprints are included in this visualization, whereas control keys are excluded.

**Figure 10:**
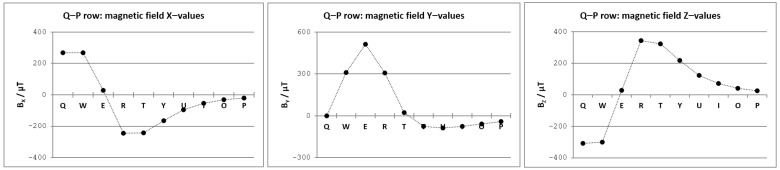
Magnetic field fingerprints for the virtual keyboard top-row (letters from Q to P).

It can be seen that larger values of the magnetic field were obtained for letter rows closer to the smartphone in a given setup. This comes as no surprise, as a permanent magnet affects the sensor readings considerably more when its position is closer to the magnetometer. By observing the obtained magnetic field curves for three letter rows, we can notice a certain regularity that may indicate the approximate position of the magnetometer in the smartphone itself. We tried to explore this regularity further by analyzing trends of $B_{x}$, $B_{y}$, and $B_{z}$ values across the letter zones in a single row (see Figure 10).

The appearance of the $B_{x}$, $B_{y}$, and $B_{z}$ graphs was the main motivating factor for the curve fitting approach. It was clear that the magnetic field vector components, acquired for the positions that reside on a straight line, could be approximated by polynomial functions.

**Figure 11:**
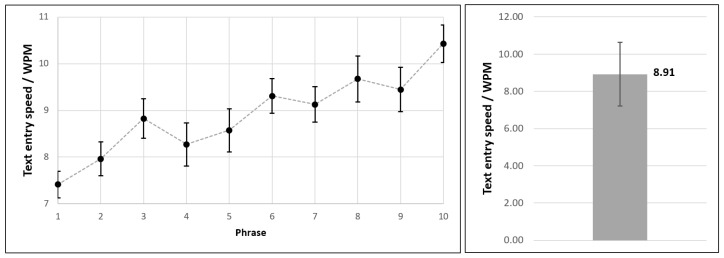
Text entry speed obtained within the empirical evaluation of magnet-based text entry method.

**Figure 12:**
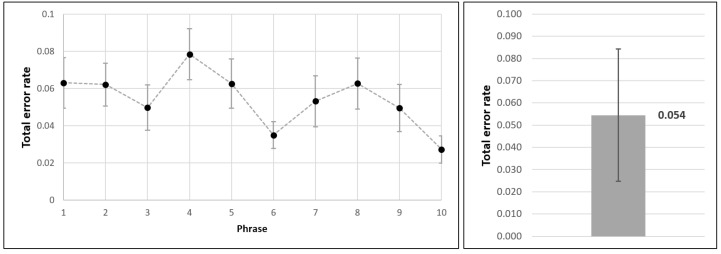
Total error rate obtained within the empirical evaluation of magnet-based text entry method.

Regarding the text entry experiment results, the participants submitted altogether 28×10=280 phrases by moving the permanent magnet across the virtual keyboard layout. We obtained all relevant text entry metrics via Text Input Logger application. Mean values and standard deviations for text entry speed (expressed as WPM) and accuracy (expressed as TER) are shown in Figure 11 and Figure 12, respectively.

First of all, we can point out the learning curve trend, as it is evident that users become more efficient over time. Namely, when typing the last phrase, they were considerably faster than at the beginning of the experiment. It should be reiterated that the users did not have formal longitudinal training prior to the actual experiment, but only a short practice session instead. Along with the increase in text entry speed, a corresponding trend of decreasing TER can also be observed.

Based on the conducted experiment, we conclude that the achieved text entry speed with the provided magnet-based input method was 8.91±1.70 WPM, with the total error rate being 0.054±0.0297.

**Figure 13:**
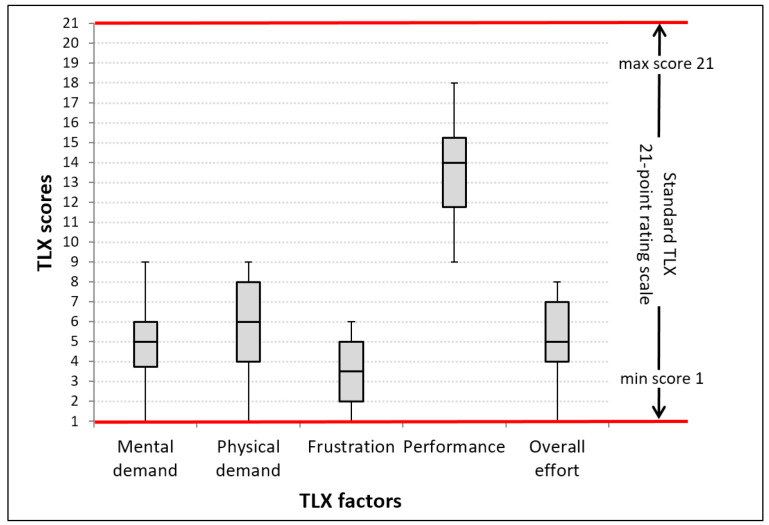
Users’ opinions on perceived workload of magnet-based text entry, obtained via paper-and-pencil version of the NASA-TLX rating scale.

As far as a qualitative evaluation of the magnet-based text entry method is concerned, the results obtained via a paper-and-pencil version of the NASA-TLX rating scale are shown in Figure 13.

Lower TLX scores for mental demand, physical demand, level of frustration, and overall effort, as well as higher scores for performance factor, indicate a better result in terms of perceived workload. It can be seen that obtained results are in line with the desired properties. Physical demand can be attributed to the need for constantly moving the permanent magnet within the virtual keyboard. On the other hand, mental demand can not be related to the character layout since the well-known QWERTY scheme was utilized. However, participants needed to master timing constraints (i.e., dwell time and delay time), which can undoubtedly affect the level of mental demand.

In general, we find the text entry evaluation outcomes, both the quantitative and qualitative ones, very promising. Text entry showed to be a good use case for providing proof-of-concept application that relies on magnetic fingerprinting.

### 3.2. Free-Move Pointing via Curve Fitting

**Figure 14:**
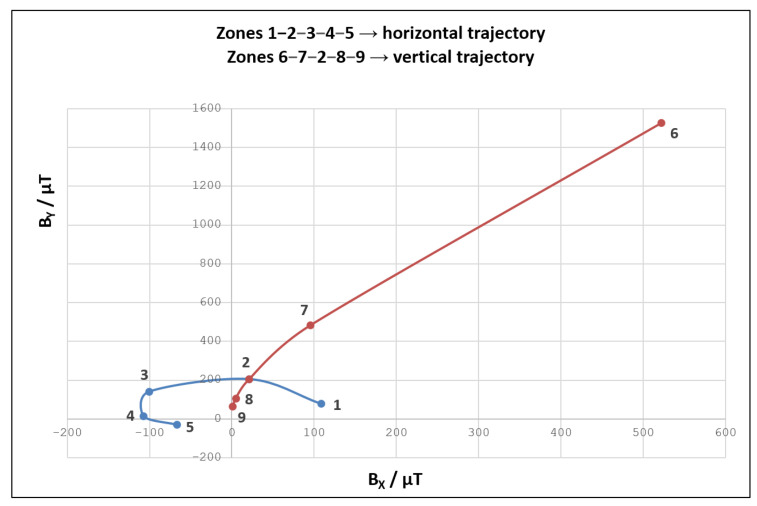
Magnetic field values obtained for 9 selected zones within the canvas utilized in the curve fitting experiment.

Canvas used within the curve fitting experiment has two highlighted trajectories, with altogether nine selected zones. Magnetic fingerprinting has been applied to these zones to construct the polynomial functions. The obtained functions are then used to predict magnetic field values along the entire length of the mentioned trajectories. The results of the described data acquisition process are shown in Figure 14.

It can be seen that magnetic field values obtained along the horizontal line (visualized in blue color) are generally smaller and less scattered than the ones originating from the vertical trajectory (visualized in red color). The magnetic field values registered for position 6 are particularly interesting because they stand out from the rest. Knowing that position 6 actually represents the starting upper point of the vertical trajectory, we can conclude that this position resides very close to the geomagnetic field sensor. Nevertheless, position 6 is still in a “safe zone”, as corresponding magnetic field values do not impose the MSO effect.

**Figure 15:**
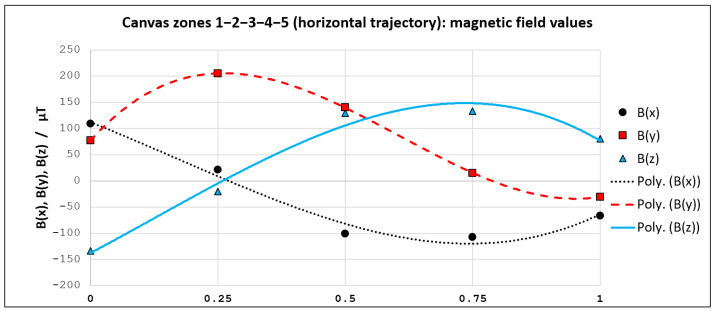
Curve fitting models for the selected horizontal trajectory.

**Figure 16:**
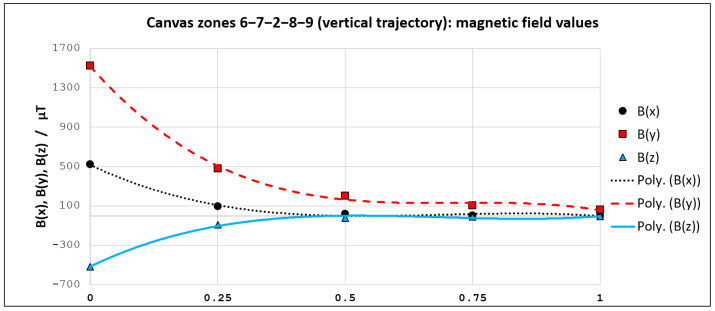
Curve fitting models for the selected vertical trajectory.

We utilized the nine obtained magnetic field fingerprints as an input for a curve-fitting procedure. $B_{x}$, $B_{y}$, and $B_{z}$ models for both trajectories have been generated with third-degree polynomials. While the related curves are depicted in Figure 15 and Figure 16, the corresponding polynomials and goodness-of-fit values are shown in Table 1. Parameter *d* in polynomial functions from Table 1 represents the discretized distance (d∈[0,…,1]) from one to the other edge of the smartphone screen.

All obtained curve fitting models have a very high goodness of fit, with $R^{2}$ ranging from 0.9801 to 0.9999. Therefore, the calculated coefficients of the polynomials have been embedded in the magnet localization algorithm (previously introduced in Figure 7a). As we wanted to provide free-move pointing along with more than just two straight lines, we utilized the same principle in order to generate eight additional curve-fitting models. Thus, for the purpose of demonstration, we modeled a total of ten trajectories (five horizontal and five vertical) for a given canvas.

We did not formally evaluate the efficiency of the localization algorithm based on the curve fitting approach. Namely, in the end, the pointer control on the smartphone display is not achieved at a satisfactory level. This is best demonstrated by the Supplementary Video File (Video S2). The possible reasons for insufficiently good pointer control are discussed in Section 4.

### 3.3. Free-Move Pointing via NNs

**Figure 17:**
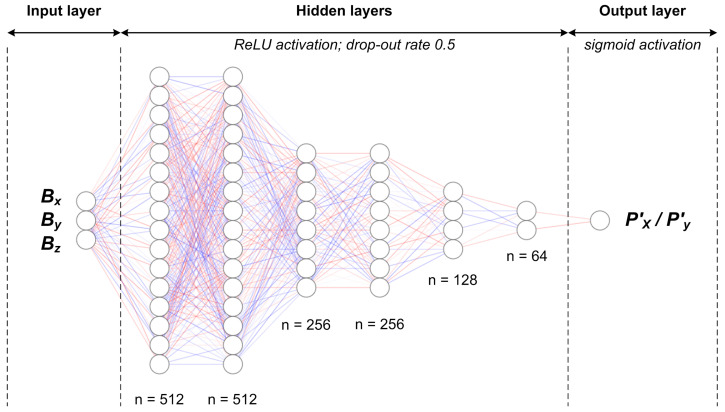
A depiction of the best performing NN topology (each hidden layer node represents 32 neurons in the real architecture).

We have depicted the best performing NN topology in Figure 17. It consists of 6 hidden layers having 512, 512, 256, 256, 128, and 64 neurons, respectively.

As mentioned before, the NN was evaluated by MSE and MAE metrics on each fold. Table 2 presents the results for each of the seven folds, as well as the average value (AVG±σ) that was taken as a trustworthy outcome when evaluating the related model. The values shown represent errors of the NNs when estimating the scale-invariant position ($P_{x}^{\prime }$, $P_{y}^{\prime }$) of the magnet. Since we have trained two NNs using the same topology, the related results are obtained for $P_{x}^{\prime }$ and $P_{y}^{\prime }$ separately. Namely, while the first model estimates $P_{x}^{\prime }$—the magnet position along the x-axis relative to the canvas width, the second model estimates $P_{y}^{\prime }$—the magnet position along the y-axis relative to the canvas height.

Since MSE makes small errors even smaller and large errors even larger, we have decided to use MAE as a metric to treat all estimation errors in the same manner. By doing so, we are actually focusing on worse outcomes because MAE tends toward a more pessimistic error value. In order to present the estimation errors more tangibly, we have translated the obtained MAE values into the coordinate systems of both the canvas grid and the smartphone display. We did so by using the canvas dimensions and the smartphone resolution as transformation factors, respectively. This procedure provided us with error values for estimating the position of the pointer ($P_{x}$, $P_{y}$) on the smartphone display, and equivalent errors for the location of the magnet ($M_{x}$, $M_{y}$) on the canvas grid. The corresponding results, expressed in physical measures (millimeters and pixels), are presented in Table 3.

**Figure 18:**
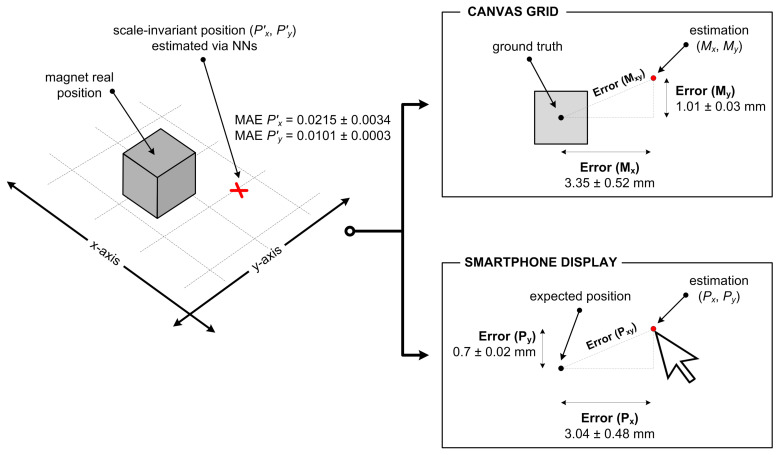
Visualization of the position estimation error, based on the obtained MAE values.

We have also introduced the values for $P_{xy}$ and $M_{xy}$ in Table 3, which reflect the combined effect of the two separate errors in the x-axis and the y-axis. These parameters represent the Euclidean distance between the pointer’s (or magnet’s) exact position and the related position estimated by the NNs. Figure 18 depicts the meaning of the data presented in Table 2 and Table 3, as well as the transformation of scale-invariant MAE values into specific position estimation errors.

Once obtained, NN models for predicting $P_{x}$ and $P_{y}$ values were incorporated into the target Android mobile application by making use of TensorFlow Lite formats. Free-move pointing was thus successfully implemented and additionally applied to simple drawing use case.

The results presented here are discussed in more detail in Section 4.

## 4. Discussion

The magnetic fingerprinting method proved to be straightforward, easy to set up, and easily adjustable to any given context. Namely, previously registered magnetic fingerprints can be simply reused in the new setting, providing that the smartphone and the dedicated canvas are in the same position as within the calibration procedure. A site-specific ambient magnetic field will not affect the interaction efficiency because only the effect produced by the permanent magnet is taken into account during calibration. If a new calibration is required for any reason, the corresponding process does not take long.

We found no particular problems in our proof-of-concept application which relies on magnetic field fingerprinting. The provided magnet-based text entry method showed to be easy to use in the augmented interaction space in front of the mobile device. Initial evaluation of text entry efficiency revealed the total error rate of 5.4%, which is in line with typical text entry systems. In other words, the obtained error rate is not a result of some shortcomings of magnet-based interaction. However, it can be attributed to the typical speed-accuracy trade-off, which is inherent in typing tasks. In terms of text entry speed, ≈9 WPM level is certainly not comparable to typing efficiency in the desktop domain, nor with the usual speeds of text input on mobile devices. However, the provided text entry method can serve as proof of the magnetic-fingerprinting-based interaction utility. In particular, by augmenting the ADI in the way shown, useful applications in the domain of assistive technology can be developed. Furthermore, applications that involve child-computer interaction and tangible user interfaces (TUIs) can be introduced as well.

As mentioned before, the curve fitting approach did not prove utterly suitable for the free-move pointing application. Namely, the achieved pointer control was not entirely smooth. Given that this approach is primarily designed to support a magnet’s movement along a straight trajectory on a virtual pane, it is not surprising that free-move pointing was not achieved at a satisfactory level. In order to get a better pointer control, the utilized localization algorithm has to deal with a considerably larger number of curve fitting models. Put differently, a large number of straight trajectories should be modeled via curve fitting for a given canvas. To put the matter into perspective, it must be noted that the related concept was demonstrated by an application that relied on ten curve fitting models. Hence, a possible solution to the described problem can be found in the high-density discretization of the given canvas. For example, for a 10 cm high canvas, we could build $B_{x}$, $B_{y}$, and $B_{z}$ models for 100 horizontal lines that are 1 mm apart. In that way, the corresponding algorithm would look for the best position candidate among a larger number of built curve-fitting models. As a result, the magnet localization, and thus the pointer visualization on the smartphone screen, should be more precise. However, such an approach practically implies a hard-coded solution, which we a priori dismissed as undesirable.

According to the aforementioned, we can argue that the curve fitting approach is not entirely suitable for free-move pointing when the magnet changes its direction very often. Nevertheless, it can indeed find its application in cases where it is necessary to detect and adequately utilize the rectilinear movement of the magnet. Typical elements of user interfaces that can be operated in the described manner are scroll handles and sliders. These are commonly used for content scrolling, playback volume control, screen brightness control, audio and video fast-forwarding, etc.

Unlike the curve fitting approach, the utilization of the NN models provided satisfying results on free-move pointing. For magnet position estimation, we obtained MSE of 0.001±0.0004 for the x-axis, and 0.0003±0.0 for the y-axis. The associated MAE values, which we consider more relevant in our case, are 0.0215±0.0034 for the $P_{x}^{\prime }$ coordinate, and 0.0101±0.0003 for the $P_{y}^{\prime }$ coordinate. The estimation errors are much higher for the *x* component, which is a result of the fact that the related *x* coordinate refers to the wider side of both the smartphone screen and the canvas grid. Namely, the display dimensions of the utilized device are 141.6×68.9 mm, assuming the landscape orientation as the default in the given context. The resolution of the smartphone display in such an orientation is 2960×1440 pixels. The corresponding dimensions of the canvas grid, on which the magnet was being continuously moved, are 155×100 mm.

When the scale-invariant MAE values are applied to the smartphone and canvas coordinate systems, the effects of NN estimation errors can be more easily assessed. Namely, in terms of physical representation, the obtained estimation errors for pointer’s coordinates $P_{x}$ and $P_{y}$ are 63.5 pixels (=3 mm) and 14.6 pixels (=0.7 mm), respectively. We can argue that this level of pointer positioning error cannot affect the usability of the free-move pointing considerably. As for the position of the permanent magnet on the given canvas grid, it is also estimated with an acceptable level of error (3.5±0.5 mm for a 2D context). We observed that larger localization errors generally occur when the magnet is positioned further away from the smartphone device.

As shown in the provided Supplementary Video File (Video S3), the effect of localization error within the NN approach is negligible. Namely, the pointer in the mobile application can be controlled smoothly. In the same video file, we demonstrate a simple drawing use case, which relies on free-move pointing. Therefore, the NN approach can indeed be successfully used for providing pointing via magnet localization, based only on the average $B_{x}$, $B_{y}$, and $B_{z}$ readings of the smartphone’s built-in geomagnetic field sensor.

Regarding the comparison of the obtained results with those originating from similar papers, it is worth noting the most commonly used types of outcomes. Namely, the related researches either: (1) Present proof-of-concept prototypes without quantitative indicators, or (2) mainly report the accuracy of magnet-based gesture recognition. For example, if a magnetic ring is worn on the finger, Magstroke [21] system can detect keystroke gestures with an accuracy of 95%. Similarly, MagiTact [13] solution can identify in-air hand gestures with an accuracy greater than 90%. MagController [16] can recognize the magnetic actions with an accuracy of 97% in different magnet’s orientations relative to the device. However, our measurable outcome is not the gesture recognition accuracy but, in the free-move pointing case, the accuracy of magnet localization in real-time. In this context, the TMotion [24] prototype, with existing data on the accuracy of magnet tracking in 3D, represents one solution with which we can meaningfully compare. Namely, TMotion can estimate the magnet’s position above the mobile device, within a 160×160×100 mm 3D-space, with an average localization error of 6.27±4.56 mm. In our case, within a 155×100 mm 2D interaction space, the magnet’s position within the canvas grid is estimated with an error being 3.5±0.5 mm. We can argue that these results are quite comparable and that the smaller error in our case is due to the 2D context only. However, let us recall that, unlike our solution, TMotion uses a specially designed stylus that incorporates both a permanent magnet and an IMU device. As for the efficiency of the magnet-based text entry, to the best of our knowledge, there is no related work that reports WPM and TER metrics for a similar method.

The provided free-move pointing could be furthermore investigated, so as to support 2D stroke gesture recognition. For this purpose, gesture recognizers that are independent of the coordinate system, such as !FTL [42], could be used. Moreover, instead of translating the magnet location into a pointer position, its movement on the nearby surface could be interpreted for manipulating contents displayed on the smartphone. In this way, tangible interaction would be supported, with physical objects that are light and easy to handle.

## 5. Conclusions

In this paper, we demonstrated how the interaction space around the mobile device could be successfully augmented by intentional distortion of the ambient magnetic field. Namely, a strong permanent magnet is used to manipulate the smartphone’s built-in sensor readings, which are interpreted as the corresponding interaction commands. We have presented and applied different approaches for supporting magnet-based interaction, including the simpler ones (magnetic field fingerprinting), as well as the more complex ones (NNs). We have shown that even moderately-complex models are capable of attaining highly functional interaction.

We provided two proof-of-concept applications that incorporate magnet-based interaction: (1) Text entry enabled by discrete magnet positioning and (2) free-move pointing based on continuous magnet movement. The related solutions eliminate the need for direct physical contact with the touchscreen and do not require alterations in device hardware or sophisticated peripherals. The provided magnet-based text entry method was initially assessed by conducting a controlled experiment, the promising results of which served as a motivation for further research. To support free-move pointing, we tried to take advantage of the curve fitting approach; however, it fared worse compared to the NN approach.

Our future work plan consists of addressing the limitations concerning how the magnet has to be moved in front of the mobile device. Namely, we want to investigate the possibilities to mitigate the strict condition for keeping the magnet in the same orientation. Furthermore, we plan to formally evaluate magnet-based free-move pointing efficiency by conducting the experiment based on pointing tasks defined in ISO 9241-411 standard [43]. The preparations for these activities are already underway.

## Figures and Tables

**Table 1:** Polynomial functions and related goodness-of-fit values for the selected trajectories in the curve fitting experiment.

Trajectory	Magnetic Field Component	Curve Fitting Model (Polynomial)	$R^{2}$
horizontal	$B_{x}$	$435.2d^{3}-228.03d^{2}-382.79d+112.06$	0.9804
	$B_{y}$	$1453.3.2d^{3}-2644.9d^{2}+1084.3d+76.906$	0.9999
	$B_{z}$	$-488.64d^{3}-187.54d^{2}+515.48d-137.71$	0.9801
vertical	$B_{x}$	$-1812.5d^{3}+3751.1d^{2}-2459.4d+518.41$	0.9955
	$B_{y}$	$-3793.1d^{3}+8180.4d^{2}-5849.6d+1518.7$	0.9979
	$B_{z}$	$1859.8d^{3}-3831.6d^{2}+2483.3d-514.16$	0.9951

**Table 2:** Results of NN training for magnet position estimation (estimation error is relative to the canvas/display dimensions).

Fold	MSE $P_{x}^{\prime }$	MAE $P_{x}^{\prime }$	MSE $P_{y}^{\prime }$	MAE $P_{y}^{\prime }$
Fold 1	0.00142	0.01840	0.00027	0.00967
Fold 2	0.00142	0.01840	0.00027	0.00967
Fold 3	0.00142	0.01840	0.00027	0.01031
Fold 4	0.00076	0.02596	0.00027	0.01031
Fold 5	0.00076	0.02596	0.00027	0.01031
Fold 6	0.00065	0.02150	0.00027	0.01034
Fold 7	0.00065	0.02150	0.00027	0.01034
**AVG** ± σ	0.0010 ± 0.0004	0.0215 ± 0.0034	0.0003 ± 0.0	0.0101 ± 0.0003

**Table 3:** Estimation errors of the NN models (based on MAE values), expressed in physical measures, for both the smartphone pointer position and the magnet location.

Smartphone	Error $\left(P_{x}\right)$	Error $\left(P_{y}\right)$	Error $\left(P_{\mathrm{xy}}\right)$
Pointer position (pixels)	63.5 ± 10.0	14.6 ± 0.5	65.2 ± 9.9
Pointer position (millimeters)	3.04 ± 0.48	0.70 ± 0.02	3.12 ± 0.47
**Canvas grid**	**Error ** $\left(M_{x}\right)$	**Error ** $\left(M_{y}\right)$	**Error ** $\left(M_{\mathrm{xy}}\right)$
Magnet location (millimeters)	3.35 ± 0.52	1.01 ± 0.03	3.50 ± 0.50

## Data Availability

Not applicable.

## Supplementary Materials

The following are available online at https://www.mdpi.com/article/10.3390/s21093087/s1, Video S1: Text_entry.mp4; Video S2: curve_fitting.mp4; Video S3: nn.mp4.

## Abbreviations

The following abbreviations are used in this manuscript:

ADI	Around-Device Interaction
NN(s)	Neural Network(s)
VR	Virtual Reality
IMU	Inertial Measurement Unit
CNN	Convolutional Neural Network
MSO	Magnetic Sensor Overflow
IME	Input Method Editor
WPM	Words Per Minute
TER	Total Error Rate
TLX	Task Load Index
MSE	Mean Squared Error
MAE	Mean Absolute Error
TUI	Tangible User Interface
